# “The Efforts” of the Dental Cosmos to “Render Justice,” &c.

**Published:** 1864-07

**Authors:** B. Wood


### "The EFFORTS" OF THE DENTAL COSMOS TO "RENDER JUSTICE," &C.

BY B. WOOD, M. D.

In the April number of the *Dental Cosmos* appeared an editorial by Dr. J. D. White, ostensibly with a view to answer letters in relation to the Plastic Metallic Filling, wherein the writer makes an effort to prejudice this material, and to damage the proposer of it in interest and reputation, as much as possible. Its sneering caption foretells its animus. It is written in bad temper—indeed quite "bilious." It betrays an incurable prejudice from the first, and reveals an early formed *intention* of avoiding any direct mention of the material in question as long as could be, and preparation for assailing it whenever it came up in a "way" that made it "necessary" to come out openly. It proposes to be in "answer" to a brief note, with sample of material received from me nearly a year ago, and which is held up as the occasion of the present writing, although it exhibits some attempts at reply to the paper on "The Disposition made of Communications," while avoiding any mention of it. It comes in "questionable shape" but its object is unmistakable.

As to the material in question, the writer opposes no facts or arguments, and adduces no disproof of what has been said of it or testified in it its favor; simply he does "not find use for it *because*" he does not use it! and then proceeds to hold it up as being an old *mercurial compound*, such as he used under the name of "*White's* Terra Metallic Compound," a "quarter of a century ago!" It is fortunate that these efforts to render justice should have become patent, that they may be duly appreciated by the profession.

I have presented a reply to this article elsewhere,* and only refer to it here, because of its relevance to the present subject.

The above mentioned article from the Editor of the *Cosmos* in the April number, is followed up in the May number by an article from the publisher, which is remarkable for the fidelity with which it "follows in the footsteps of its illustrious predecessor." This takes for its text the communication in the March number of the *Dental Register* on "Disposition made of Communications," and which, as is conceded, "argues the duties of Journals to writers and readers; but the *argument* is totally ignored, and the whole

* *Dental Times*, July 1864.

paper devoted to personal matter. The drift is to *the effect* that said communication was personally "against the publisher" for refusing longer to publish, as communications, a repetition of matter designed for advertisement, in regard to a subject with which the profession had long ago become thoroughly acquainted. That his generosity in the matter had been already pressed too far. That he had at last stopped it once for all. That this was merely a matter of *personal complaint* on my part. That on the contrary I had "no *just* cause of complaint," but rather for gratitude for favors and condescension shown; all of which is backed by the presentation of a list of articles which had been published in the *Cosmos* in relation to the Plastic Metallic Filling. But the pith of all would seem designed to lend confirmation and endorsement to Dr. J. D. White's article, and to proclaim the "unfavorable verdict which HE "has pronounced" against the material.

Now, I do not propose in this place to come out in personal defense against inuendoes and untangible issues. But there are certain principles of justness, truth and sincerity for which I desire to elicit due respect. There is a path which all should aim to follow, and if we deviate therefrom, unwittingly or willfully we should be led back by strong or gentle pressure, as exigencies require.

As to the communication referred to, whether a "complaint" or a discussion of principles, it is before the profession. For its fairness and temper it is amenable to just criticism. It is what I should have *written* to Dr. J. D. or S. S. White, or any friend, if supposed to be wrong and yet open for correction. But there are cases in which judgment and consciousness can not be reached except in such a way as shall bring public sentiment to bear.

In proceeding to his details, Dr. White adroitly evades the argument, by turning it into a personal issue. Let us see where he leads to at this.

Having quoted a sentence which concludes by saying, that

"one statement of the same information is all a writer can ask, and all that a journal promises to give, unless it be for the elucidation of new matter;" he says, "that Dr. Wood has no just cause of complaint, judged by his own standard, will appear from the following exhibits:" whereupon follows the "exhibit" of articles purporting to relate or reiterate information as to the "Plastic Metallic Filling," making out quite a formidable account and plausible case in the Doctor's favor; and in conclusion he expresses satisfaction with "the efforts to render justice to Dr. Wood and to the profession." Let us now take them in detail, and see if the natural tact and pains-taking displayed in drawing them up ought not to be included among the said "efforts."

Exhibit, No. 1. "An article was copied from the *Chemical News*, in the Periscope of the *Dental Cosmos* for May 1861, page 579, entitled Wood's Fusible Metal.

This it is to be remarked, was before the Plastic Metallic Filling" was produced, and it is about as much to the purpose as though the notice related to "Babbitt's Metal." This notice quoted from a foreign journal (the *London Chemical News*,) of a discovery made by an American dentist, and announced by our own scientific journals a year before, is due to the medical gentleman who has charge of the Periscope department of the *Cosmos*.

No. 2. "In the same department of the *Dental Cosmos* for Feb., 1862, p. 401, copied from the *Franklin Institute Journal*, Dr. Wood's own language is given [!] occupying more than two pages." [!] I hope my very gracious friend intends no spite in nicknaming a scientific journal which has published many pages from the same source, and paid for them, without subsequent reflections. But to what did that—wherein the "OWN LANGUAGE *was given, occupying*" *so much room*—relate. Not to the "Plastic Metallic Filling" (which was not yet announced) nor to "Wood's Fusible Metal," described two years before. But to a new and different discovery, remarkable in a scientific view, but of no practical interest in operative dentistry. The article was entitled "New Fusible Alloys:—Remarks on Determining the Melting Point of Metals,"—two-thirds of the paper being devoted to the latter topic—(which, by the way, might be worth attending to by certain experimenters.)

This is the sum of what even the "exhibit" *pretends* to have communicated about "Wood's Filling" prior to the "communications direct from himself" below referred to.

No. 3. "In the Review department," January, 1863, "copied from the *Dental Register* an article from Prof. Taft, highly eulogistic of Dr. Wood's invention; and"—[to neutralize the same?]

No. 4. "At page 328, same number, an unfavorable report of a trial of it, taken from the *American Dental Review*."

Prof. Taft's article, so "highly eulogistic" of a thing condemned upon "a trial of it" by the stately "*Review*," detailed his method of applying the Filling in cases where it had not been recommended, concluding by expressing himself "much pleased with the cases thus far." The other (exhibit No. 4,) refers to an *anonymous* article in a small advertising pamphlet (with a large name) issued occasionally, (and but once within three years); which reported, not "a trial" but "an experiment" wherein the writer condemned the material *without trial.* It was copied in the *Cosmos* "before the ink was dry." This has been referred to, pointing out the error in the experiment, not to take exceptions to the writer, for the spirit to *examine* for one's self is sufficient to atone for some blunders—although "justice to Dr. Wood and to the profession," required that the correction, when made, should have been as promptly copied.

But to proceed:—In the way of communications direct from himself, relating to the material under discussion there was published."

No. 5. *Dental Cosmos* for September, 1862, page 59, "Plastic Metallic Filling for Carious Teeeth:"

"This was the only communication upon this material submitted by me to the *Cosmos*, except the one describing the *manner* of *using* it, which was "respectfully declined," although the material was referred to in one or two other articles. It comprised four and a half pages, a large portion taken up with the history and character (quoted from the older authors) of the old fusible metal and mercurial compounds formerly used, and such as Dr. J. D. White, with this light before him, seeks to identify with the Plastic Metallic Filling.

No. 6. Feb., 1863, page 353, "Materials for Filling Teeth," (read before the Brooklyn Dental Association.)

This consisted of some eight or nine printed pages, the result of much previous study and comparison, and, except the last page, could have been written by me or any one else, with the same examination, had the Plastic Metallic Filling not been in existence; the major part of what is descriptive of this had been omitted in the revision for publication, as the essential points had already been published, and the rest was optional with the *Cosmos*, under the standing request to publish nothing submitted unless of *general interest to the profession.* Have the facts as stated been controverted? or, if what was said in relation to oxichloride of zinc gave offense, was it not indisputable and fairly stated?

No. 7. "These two articles made thirteen pages, which were not only published, but for which Dr. Wood was paid as a contributor."

Is it unusual, then, for the *Cosmos* to pay for communications? I have been under the impression it was a standing inducement held out. In my own case it certainly was. Is it then a mere lure? or a pretense of superior value for what appears in the *Cosmos?* It is well, perhaps, to have put it to test, since it had not been before. And then, Dr. White was left legally free to pay or not to pay, or have it back if dissatisfied. But I have *since* understood that *papers read before Societies* are ruled out of the pay list. If the previous reading of a paper to half a dozen dentists or more, met as a society, cancels the bill, the *Cosmos* may hold out sounding inducements and yet have *very little to pay for.*

Dr. White intimates that it was a great and special condecension. The Doctor is stalking quite largely—ON STILTS—keeping very equal pace with his predecessor. Although stilts may answer very well for getting over muddy places, where there is sound bottom, they do not serve so well for extrication in a quagmire.

Doubtless those journals devoted to general science, which offer their columns for the announcement of inventions and discoveries, without stopping to consider whether the facts communicated might not advantage the writers, and if it is their custom to pay contributors, *do so*, uninfluenced by such considerations, will be regarded as quite small affairs by the side of the *Cosmos*, wholly unacquainted with the "rules" of journalism and their "duties" to writers and readers,"

[No. 8. ?] In the last article published in the *Dental Cosmos* for Feb., 1863, page 360, speaking of the "Plastic Metallic Filling," &c.

Now this is singularly worded: — it refers to the article just above enumerated. There were not two articles published in *Cosmos* for February. Neither was the one above mentioned the "last article published in the *Dental Cosmos*" on the subject. There was another subsequently published—The paper read before the American Dental Convention published in the *Cosmos*, September, 1863—) which it is singular Dr. White should have abstained from reckoning in the count, since in chiefly setting forth the *advantages* of the material, and even stating the conditions of sale, it really partook of the nature of "an advertisement,—being the only one that could "properly" be classed under that head. However, the proof was too ready that this was not communicated to the *Cosmos*, but objections to publication in it were only partially waived, after protest, upon assurance that it was a part of the proceedings of the convention, in regard to which the conductors of the *Cosmos* had nothing to do. (When taken by one of the Reporters, after the reading, I did not know at the time whether the Reporter of the *Vulcanite* or the *Cosmos*). Perhaps it will be considered wonderful by some, that any one should object to "figuring in the journals," especially when his "interest" might be subserved. And to be prudish in such matters is certainly no better than to be overweening. But it is not a universal trait to court such things, by self-humiliation. A reasonable independence will often forego many important objects of desire if patronizingly bestowed. Dentists as practitioners all desire reputation and patronage, but do not court the class of patients who are always impressing a sense of favor conferred, especially if there is enough else to do. This is natural in respect to journals also. If the contribution in the Eebruary number was not "the *last*," the *Cosmos* probably was not unaware that it was *intended* to be. Why, then, was another submitted? (It is a private affair, but it has a "moral as well as a practical meaning.") In February, 1863, Dr. White thought the profession had already become sufficiently acquainted with the subject, and therefore, in September following, declined to publish anything more about it. In August he appeared to see things differently. For although up to that time, (as he informed me after the adjournment of the convention) he himself "had not confidence in the material—did not believe it practical—and therefore had never recommended it; but had said nothing against it;" yet, "to see the high testimony" (as he expressed it) there elicited, appeared to have effectually removed his doubts—(to return however the succeeding month). The tone of correspondence was due to inexperienced clerks, &c. On the whole, all parties had been laboring under wrong impressions. It was in this phasis of the case that, as the profession began to take practical interest in regard to the material, and for reasons before stated, (*Register*, Jan. p. 22), "the Directions for using it were submitted to the *Cosmos*—

made as direct to the point and unexceptionable as possible, free from anything commendatory, and unencumbered with matter with which the profession were presumed to be already acquainted: — it would have occupied about two pages in the *Cosmos.*

But Dr. White, quoting from the February article, where a description of the material is waived with the remark, "It is presumed the profession have by this time become acquainted with it,"—adds:

"We thought so too, and therefore declined respectfully to publish "Directions for its use, with cuts of the instruments recommended."

If the profession had become acquainted with it, they might then desire to know something about the *manner of using* it, as well as the instruments employed. "*Therefore,* we declined," &c.

"We thought ..... and declined to publish." Who? The publisher, speaking for himself in the plural? Dr. S. S. is always punctilious to say—"Your communication has been submitted to Dr. J. D. White," and, "has been accepted by him, etc. However, in this it was a joint operation. The one "thought," and the other, after several days' "due consideration," came to the "same conclusion"—"*thought*" *for the profession*, and did not care to afford the profession further to judge for themselves. So the hapless article was doomed to the wretched fate of being "*declined* by the *Cosmos.*" And "WE" had the satisfaction!

"Declined to publish 'Directions' for its use, *with cuts of the instruments recommended.*" Professedly the same article appeared in the *Register*, but it contained no cuts, &c. A description of the instruments, with cuts, was indeed proposed, but not submitted, yet such the zeal to "render justice," in this way, as to forestall examination. I abstained from any allusion to this little matter before, but am obliged to say a word. The information proposed was at least new, and doubtless essential to a practical acquaintance with the subject; it was all that remained or was intended to be offered. Not being able to supply dealers except with sample sets, or hardly to meet the direct demands from the profession, while intending at that time to give up this charge, as soon as could be done; it occurred that a description of the instruments, with cuts, would better enable dealers and dentists to get them up for themselves. Dr. White had been supplied with patterns and was promptly responded to in regard to particular points of inquiry. Why object to the publication of similar information which might benefit others? But although it was not, as he broadly intimates, to "recommend" *my* instruments—no one pretends that the expense and trouble was without regard to remuneration, for it might have been a help in facilitating the working and general introduction of the material. But Dr. White was well advised that I did not expect or want anything published except on its own merits *in point of interest to the profession.*

But to close up the "exhibit."

No. 8. "Since then, in the November number of the *Dental Cosmos*, 1863, p. 220, Dr. McQuillen gave a favorable opinion of the article, in extreme cases, from his own pen." Was it not very condescending on the part of Dr. Mc?—and also very kind of the publisher to place him also on stilts?

It is to be remarked, that if of value in the "extreme cases" mentioned, it ought to be still more so in the ordinary run of cases.

No. 9. "In the April number, 1864, Dr. J. D. White has pronounced an unfavorable opinion." (And the publisher always comes to the same conclusion with his editor.) Whereupon Dr. S. S. expresses himself "Satisfied with the efforts to render justice to Dr. Wood and to the profession." He ought to have added the free distribution among non-subscribers of the number containing this last enumerated "effort," and then to have included this remarkable production "*from his own pen!*"—in the list.

Such, gentlemen of the profession, workers, writers, contributors, is what any of you may have to expect, should occasion offer, from some who assume to sit in judgment and to dispense "justice."

